# Molecular Characterization of Wild and Cultivated Strawberry (*Fragaria* × *ananassa*) through DNA Barcode Markers

**DOI:** 10.1155/2022/9249561

**Published:** 2022-10-11

**Authors:** Awais Qarni, Khushi Muhammad, Abdul Wahab, Amir Ali, Chandni Khizar, Izhar Ullah, Abeer Kazmi, Tahira Sultana, Asma Hameed, Muhammad Younas, Mehdi Rahimi

**Affiliations:** ^1^Department of Genetics, Hazara University Mansehra, Mansehra, Pakistan; ^2^Shanghai Center for Plant Stress Biology, CAS Center for Excellence in Molecular Plant Sciences, Chinese Academy of Sciences, Shanghai 200032, China; ^3^Department of Botany, PMAS, Arid Agriculture University, Rawalpindi, Pakistan; ^4^Institute of Molecular Biology and Biotechnology, University of the Lahore, Lahore, Pakistan; ^5^Department of Botany, University of Malakand, Chakdara, Khyber Pakhtunkhwa, Pakistan; ^6^Institute of Hydrobiology, Chinese Academy of Sciences, University of Chinese Academy of Sciences (UCAS), Wuhan, China; ^7^Department of Biotechnology, Institute of Science and High Technology and Environmental Sciences, Graduate University of Advanced Technology, Kerman, Iran

## Abstract

**Background:**

DNA barcoding is a useful technique for the identification, conservation, and diversity estimation at the species level in plants. The current research work was carried out to characterize selected *Fragaria* species from northern Pakistan using DNA barcode markers. *Methodology*. Initially, the efficacy of eight DNA barcode markers was analyzed based on the amplification and sequencing of the genome of selected *Fragaria* species. The resultant sequences were analyzed using BLAST, MEGA 7.0, and Bio Edit software. The phylogenetic tree was constructed by using *Fragaria* current species sequences and reference sequences through the neighbor-joining method or maximum likelihood method.

**Results:**

Among eight DNA barcode markers, only two (*ITS2* and *rbclC*) were amplified, and sequences were obtained. *ITS2* sequence was BLAST in NCBI for related reference species which ranged from 89.79% to 90.05% along with *Fragaria vesca* (AF163517.1) which have 99.05% identity. Similarly, the *rbclC* sequence of *Fragaria* species was ranged from 96% to 99.58% along with *Fragaria* × *ananassa* (KY358226.1) which had 99.58% identity.

**Conclusion:**

It is recommended that DNA barcode markers are a useful tool to identify the genetic diversity of a species. Moreover, this study could be helpful for the identification of the *Fragaria* species cultivated in other regions of the world.

## 1. Introduction

*Fragaria* is commonly known as wild-type strawberries, a genus of flowering plants in the *Rosaceae* family, including *Potentilla* and *Duchesnea*, which are closely related to *Fragaria* [1]. A total of seven chloroplast genomes of *Fragaria* species were completely sequenced [2]. *Fragaria vesca* whole genome has been sequenced and provides the first reference genome for *Fragaria* species and is established as the model system for *Fragaria* genome-related study [3].

Wild strawberry is native to Bhutan, Afghanistan, China, Nepal, Sikkim, Myanmar, and Pakistan [4]. Cultivated strawberry is a well-liked fruit cultivated throughout the world and most economically important processed and fresh fruits, consumed for its pleasant flavor and nutrient content, and their global production reaches 9.1 M tones in 2016; annual growth has grown over 5% in the last decade [5].

The medicinal claims of wild strawberries have been around for hundreds of years [6]. *Fragaria nubicola* plant juice is used in the treatment of profuse menstruation, tongue blemishes [7], stomach ulcers, as an antiseptic, healing of wounds, children's diarrhea, and urinary infections in different parts of Pakistan [8]. Tea is prepared from the roots and leaves of *Fragaria nubicola* and has antimicrobial and anti-inflammatory properties [4]. Traditionally, its rhizome is used to treat inflammation and contains ellagic acid, and various other glycosides, antioxidants, anti-inflammatory, anticancer agents, and anti-neurodegenerative properties are also present in *Fragaria* fruits [9].

*Fragaria* species hold promising traits for selective breeding such as abiotic-stress tolerance and acclimation or disease and pest resistance [1]. Efforts to establish a phylogenetic relationship in *Fragaria* have been hampered by the low level of variability detected in the chloroplast DNA of *Fragaria* [10]. Evidence of wild and cultivated strawberries shows that flowering and running are genetically distinct processes but mutually exclusive because the predominance of one over the other is completely linked to genetic and environmental factors [11]. Numerous species with similar morphological traits are misused as official drugs, which leads to substitution and adulteration causing issues with product safety and quality control [12].

DNA barcoding is an effective, rapid, inexpensive, and standard method for assessing and identifying various plant species [13]. In addition, this method can effectively identify unknown species or species having complex morphometric behavior [14]. This method is also used to study both interspecific and intraspecific variations. DNA barcoding was used to identify some of metazoan species by the sequences of cytochrome oxidase 1 (CO_1_) genes. DNA barcode regions, such as *rbcLc, matK, ITS2, rpoB, rpoC, rbcLc, Ycf3, ITS2,* and *trnH*-*psbA*, were used to identify flowering species [15]. According to CBOL (Consortium for the Barcode Life), the cpDNA regions (*rbclC* and*ITS*) play a crucial role in the rapid identification of plant species.

To the best of our knowledge to date, no study has been conducted to assess the genetic diversity among wild and cultivated strawberries. Similarly, no barcoding study is reported for *Fragaria nubicola* Lindl. The objectives of the current study are to estimate the genetic diversity of wild and cultivated *Fragaria* through the molecular marker and to find out suitable DNA markers linking to wild and cultivated *Fragaria* species from Mansehra, Pakistan.

## 2. Method and Material

### 2.1. Study Area and Spatial Mapping

**Figure 1:**
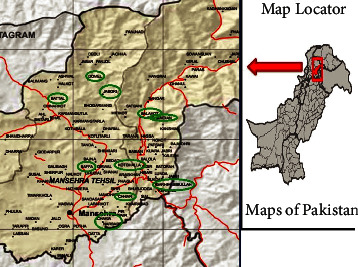
Map of district Mansehra from where genus (*Fragaria*) species were collected and the areas of Mansehra included Jabori, Garhi Habibullah, Baffa, Kund Bangla, Shogran, Batal, Balakot, Dodial, Chattar, and Qalandarabad.

The genus (*Fragaria*) species were collected from different areas of district Mansehra, Pakistan, namely, Jabori, Garhi Habibullah, Baffa, Kund Bangla, Shogran, Battal, Balakot, Dodial, Chattar, and Qalandarabad (Figure 1). Sampling areas were recorded through the help of a GPS device, from where plants were collected; at the sampling time, plant location, attitude, and elevation were recorded from different areas (Table 1).

### 2.2. Plant Collection

Plant samples were collected with the help of taxonomists. The respective samples were collected in April, May, and June 2020 in triplicate from each collection area. Suitable zipper plastic bags were used for sample collection to save it for a long duration and the best result. The plant materials were transferred to the laboratory of the genetic department, Hazara University, Mansehra, before DNA extraction. Herbarium sheets of samples were kept at room temperature in the laboratory.

### 2.3. Storage of Plant Material

The plant samples were properly dried at room temperature when plants samples were completely dried and crushed with the help of mortar and pestle. The standard scientific protocol was used for the labeling of plant tissue and then pasted on the herbarium sheet and submitted for registration.

### 2.4. Extraction of Genomic DNA

The genomic DNA was extracted from several selected plants of every position by using the modified CTAB method [16]. CTAB buffer was heated before starting the extraction of genomic DNA. We added 800 *μ*l, heated CTAB buffer (2% CTAB + tris buffer 100 ml + 1.6 g EDTA + 16.4 g NaCl+ 0.3%BME +2.4 g PVP+ 100 ml water) in each tube, and incubated at 56°C for day and night. Then, 500 *μ*l PCI (phenol chloroform iso-amyl alcohol at a ratio of 24:1) was added and centrifuged for 20 minutes at 8,000 rpm. The upper layer (supernatant) was transferred to new fresh labeled tubes. Then, we added 500 *μ*l cold iso-propanol (C_3_H_8_O) and mixed well until DNA precipitated. Tubes were spun for 15 minutes at 8,000 rpm, and the supernatant was discarded. The pellet was washed by adding 70% ethanol into each tube and centrifuged for 5 minutes at 10,000 rpm. The ethanol was discarded from tubes, and the tubes were kept at room temperature for drying. Then, we added 60 *μ*l ddH_2_O in each tube to dilute genomic DNA.

### 2.5. Selection of DNA Barcode Markers

Different DNA barcode markers were used to amplify the desired fragment in the genome of selected *Fragaria* species DNA and checked the different barcode markers by using a specific sequence of DNA, namely, *ITS2, matK, rbcL, ITS, rbclA, rbclC, Ycf3,* and *trnV* (Table 2). The barcode markers were selected on the basis of previous literature related to *Fragaria* species [24–26].

### 2.6. Gel Electrophoresis

The quality of isolated genomic DNA of *Fragaria* species was examined and checked through 1% agarose gel electrophoresis.100 ml of distilled water was taken in the flask, and we dissolved 1 gram of agarose powder and added 2000 *μ*l 50XTAE buffer and shook it well. We put the flask in the microwave for 1 minute and then kept the flask at room temperature to solidify. We added 25 *μ*l of ethidium bromide before solidifying the solution of agarose gel. Then, we transferred the prepared solution to gel tray and put combs for production of wells. The combs were removed after solidification of gel, and then gel was transferred to a gel tank. Gel tank had 600 ml of 1XTAE buffer. 5 *μ*l of genomic DNA and 2 *μ*l of dye were loaded in each well of gel. Run the gel for around 30 minutes at a voltage of 80 volts. Then, gel was run on UV lights to check the quality of genomic DNA and for documentation of the gel image.

### 2.7. PCR Reaction Reagents

The amplification of targeted DNA barcodes in the genome of selected *Fragaria* species was amplified using (Thermo Fisher Scientific, #F-548S) and followed the manufacture protocol. To obtain the target DNA fragment, the prepared PCR reaction mixture was placed in the ABI thermocycler, and the standard optimized program was adjusted in the mixture. The amplified PCR products were further confirmed by 1.5% agarose gel; gel photographs were taken in the UV apparatus (Table 3).

### 2.8. Sequencing and Phylogenetic Analysis

The obtained PCR results were correctly labeled for commercial sequencing and sent to BGI (Beijing Genomic Institute) for nucleotide sequencing. Later, the successful sequences were further analyzed by using BLAST (Basic Local Alignment Search Tool) at NCBI (database), Bio Edit, and MEGA 7.0 software for the construction of the phylogenetic tree. The phylogenetic trees were constructed of the investigated sequences through different methods (viz. maximum likelihood (ML), neighbor-joining (NJ), and maximum parsimony (MP) methods).

## 3. Results and Discussion

The Rosaceae family is a diverse and widely spread family of flowering plants, consisting of 91 genera and around 3000 species. Members of the Rosaceae family are perennial, herbs, and shrubs and native to northern temperate and tropical countries [27]. The biologically important plant species must be identified and evaluated, which plays an important role in understanding the evolutionary history of plant species.

**Figure 2:**
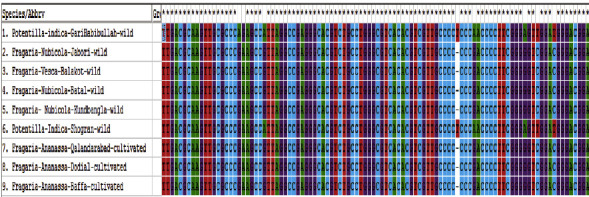
Alignment of *Fragaria* species of the *ITS2* marker.

**Figure 3:**
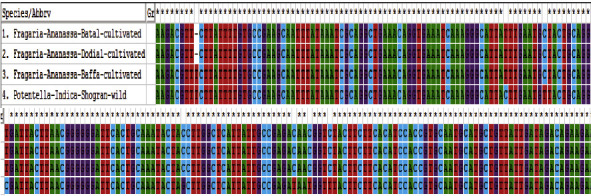
Alignment of *Fragaria* species of the *rbclC* marker.

This study shows that DNA barcoding is an efficient tool for species identification for many taxonomic groups of vascular plants. DNA barcoding is a better technique for distinguishing species. The DNA barcoding technique offers the way to study the phylogenetic relationship and genetic diversity between species populations based on short conserved nucleotide sequences of the genome [28, 29]. An expert taxonomist can solve the problem by using the DNA barcoding technique when the known species do not closely match the unknown species. In this study, the DNA barcoding markers such as *ITS2, matK, rbcL, ITS, rbclA, rbclC, Ycf3,* and *trnV* were applied against the high quality of *Fragaria* species DNA. Out of these markers, only two markers (*ITS2* and *rbclC*) were successfully amplified, and sequences were obtained from BGI (Beijing Genomic Institute) China (Figures 2 and 3). The *ITS2* marker showed 64% suitability, and *rbclC* showed 28.57% suitability against investigated *Fragaria* species.

**Figure 4:**
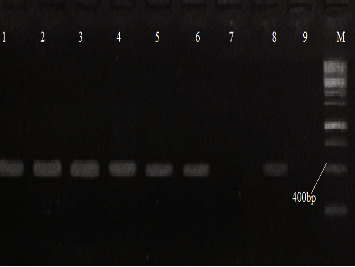
Amplified PCR products of selected *Fragaria* species of the ITS2 marker and 1 kb DNA ladder.

The large scale phylogenetic exploration of *Fragaria* (strawberry) species was analyzed through 454 sequencing platforms and the Fluidigm access array method and then found the discrepancy among two wild octoploid species, i.e., *F. chiloensis* and *F. virginiana* [30]. In the current study, the genomic DNA of selected samples was extracted, and amplification of the targeted barcode region in the genome of selected *Fragaria* species was carried out through various PCR conditions. The amplified PCR products of the selected *Fragaria* species of the ITS2 marker can be seen in Figure 4. In a previous study, Potter et al. reported that on the *ITS* marker, *Fragaria nubicola* (AF163517) showed similarity with *Fragaria vesca* (AF163485), and *Fragaria* × *ananassa* (AF163494) showed similarity with *Fragaria virginiana* (AF163479). On the *trnL* marker, *Fragaria nubicola* (AF163562) showed similarity with *Fragaria vesca* (AF163542) and *Fragaria* × *ananassa* (AF163538) [31]. While in the current study, *ITS* and *trnL* markers were found unsuitable to determine diversity. Similarly, in a previous report, *Fragaria* × *ananassa* (JX117907) showed similarity with *Fragaria chiloensis* (JX402801) on the *ycf3* marker through the maximum likelihood method [32], but no results were obtained by using the *ycf3* marker in the current study. Potter et al. reported that *Fragaria vesca* (AF288102) showed similarity with *potentilla anserine* (AF2881113) through maximum parsimony on the *matK* marker [33], while no results were obtained on the *matK* marker as well in the current study.

### 3.1. Molecular Phylogenetic Analysis of *Fragaria* by the *ITS2* Marker

DNA barcoding, which utilizes the ribosomal DNA ITS2 region as a tag to identify species, has gained a lot of attention recently [34]. ITS2 has several benefits including strong universality, low intraspecific variance but significant interspecific divergence, and a short fragment length (200 bp) over other suggested DNA barcodes such as psbA-trnH, matK, rbcL, and ITS [35–37]. DNA barcoding technology is used to identify the known as well as unknown species of berry fruit products. It provides much accurate information regarding species which is to be recognized [38]. In a recent study, approximately 37 species of *F. nilgerrensis* belonging to five diverse genetic groups were identified through molecular markers which further helped in identification, classification, and evaluation of its whole genetic pool within germplasm [39]. To the best of our knowledge, this is the first time that the ITS2 regions have been utilized to identify *Fragaria* species.

**Figure 5:**
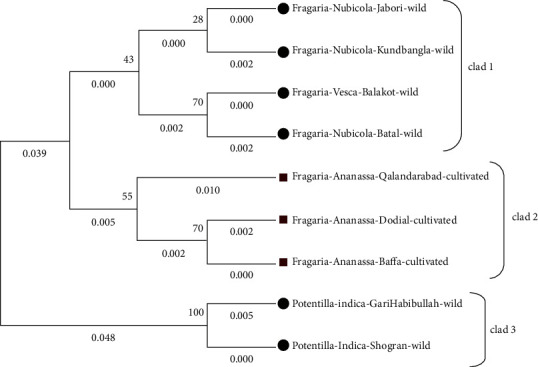
Molecular phylogenetic tree of wild and cultivated species of *Fragaria* with the *ITS2* marker through the maximum likelihood method.

**Figure 6:**
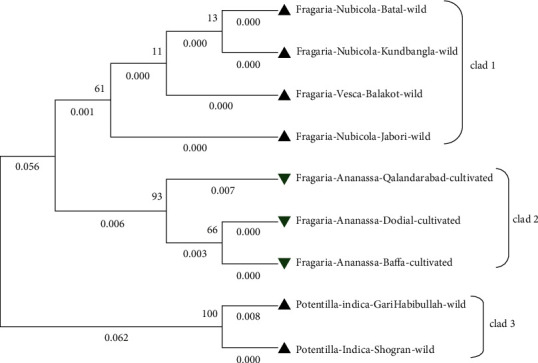
Phylogenetic tree among wild and cultivated *Fragaria* species of the *ITS2* marker through the neighbor-joining method.

**Figure 7:**
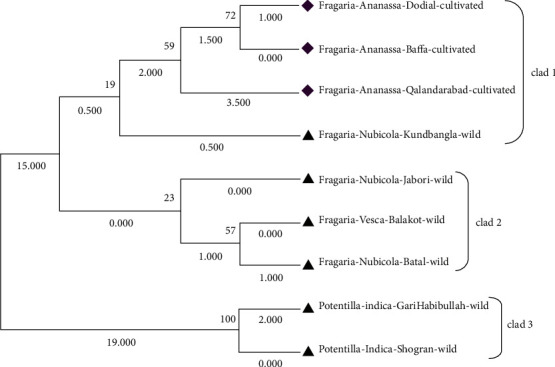
*ITS2*-based phylogenetic tree among wild and cultivated *Fragaria* species through the maximum parsimony method.

The molecular phylogenetic analysis of *Fragaria* of the *ITS2* marker was conducted through different methods. The phylogenetic tree was constructed using the bootstrap mode among wild *Fragaria* and cultivated *Fragaria* species through three different methods. The tree was distributed into 3 clades. *ITS2* sequence was BLAST in NCBI (database) for related reference species which ranges from 89.79% to 99.05% along with *Fragaria vesca* (AF163517.1) which have 99.05% identity and 1e.517 E value and 69% query coverage. In all phylogenetic trees, the wild-type *Fragaria nubicola* Lindl from Jabori, KundBangla, Battal and *Fragaria vesca* from Balakot were together in clad 1. Cultivated *Fragaria* × *ananassa* Duch from Dodial, Qalandarabab, and Baffa was placed in clad 2. In the 3^rd^ clad, *Potentilla indica* from Shogran and Garhi Habibullah was positioned together with 0.048 branch length (Figures 5–7). The phylogenetic evolution of ten wild species of *Fragaria* was identified through complete chloroplast genome sequencing by clustering *Fragaria* species into two clades and explored their common ancestor and divergent species among them [40].

**Figure 8:**
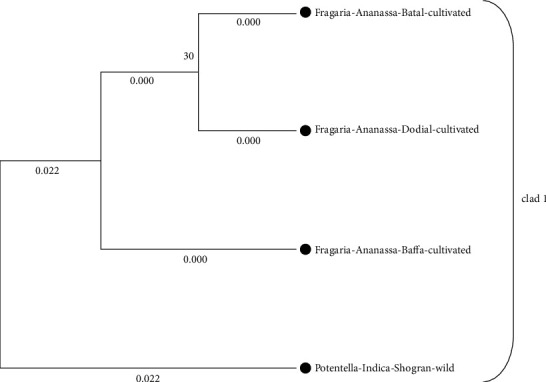
Phylogenetic tree of cultivated *Fragaria* species sequences of (*rbclC*) through the maximum likelihood method.

**Figure 9:**
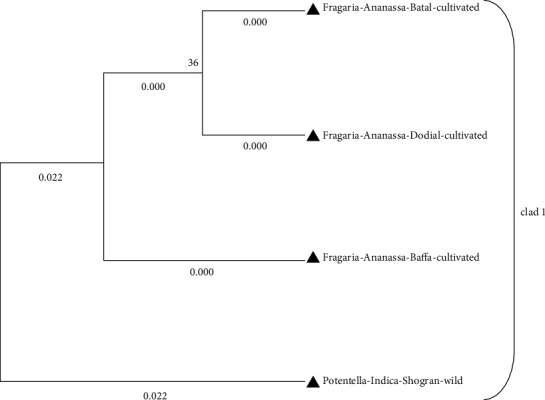
Phylogenetic tree of current *Fragaria* species sequences of *rbclC* through the neighbor-joining method.

**Figure 10:**
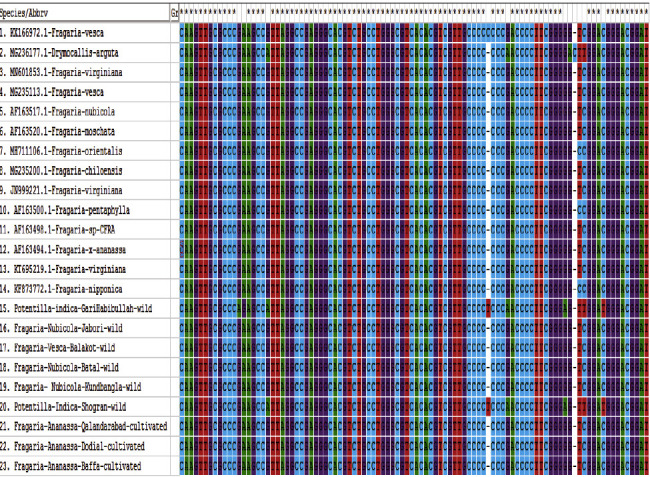
Alignment of *Fragaria* species sequences by the *ITS2* marker with related reference species.

Phylogenetic analysis of *Fragaria* species through the neighbor-joining method revealed that one clad was formed in each linkage tree. In clad 1, *Fragaria* × *ananassa* from Battal, Dodial, and Baffa was together while *Potentilla indica* from Shogran showed diversity but due to low bootstrap value and branch length (0.022), and it is also placed in the similar clad (Figures 8 and 9). On the parsimony method, there are no parsimony informative sites. The sequences were got in the FASTA format and used for alignment through MEGA 7 (Cluster W) software. The selected *Fragaria* sequences and reference sequences were aligned and trimmed by removing the irregular sequences and then used for molecular phylogenetic analysis (Figure 10).

**Figure 11:**
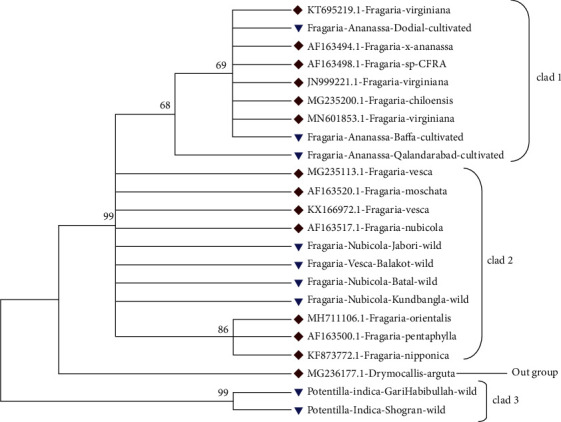
Phylogenetic tree of *Fragaria* species of (*ITS2*) with related reference *Fragaria* species through the maximum likelihood method.

**Figure 12:**
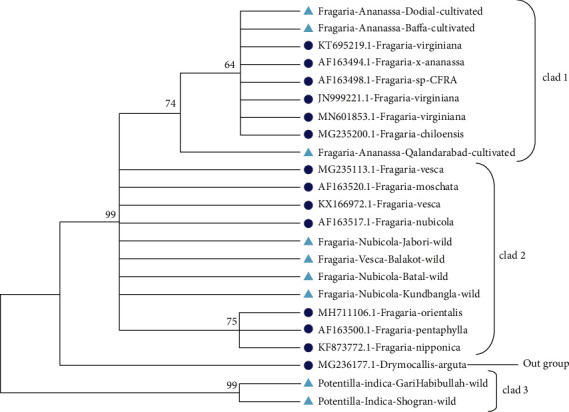
Phylogenetic tree of selected *Fragaria* species (*ITS2*) with related reference *Fragaria* species through the neighbor-joining method.

Similarly, for phylogenetic analysis of *Fragaria* species with related reference species, the phylogenetic tree was constructed through the maximum likelihood method by using the bootstrap mode and the highest log-likelihood which are -630.3100. In clad 1, *Fragaria* × *ananassa* Duch from Dodial, Qalandarabad, and Baffa and the reference species (KT695219.1, AF163498.1, JN999221.1, MG235200.1, and MN601853.1) were together. *Fragaria nubicola* Lindl from Jabori, Battal, KundBangla, *Fragaria vesca* from Balakot, *Fragaria nubicola* (AF163517.1), *Fragaria vesca* (KX166972.1, MG235113.1), *Fragaria moschata* (AF163520.1), *Fragaria orientalis* (MH711106.1), *Fragaria pentaphylla* (AF163500.1), and *Fragaria nipponica* (KF873772.1) were together in clad 2. The *Potentilla indica* from Shogran and Garhi Habibullah were together in clad 3, while *Drymocallis arguta* (MG236177.1) was placed out of the group (Figures 11 and 12).

**Figure 13:**
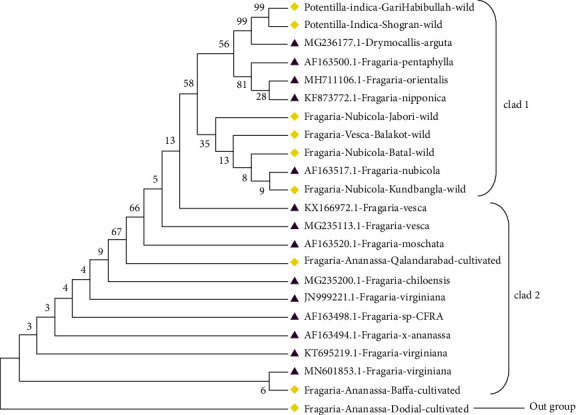
Phylogenetic tree of selected *Fragaria* sequences of the ITS2 marker with reference sequences by using the maximum parsimony method.

Phylogenetic tree analysis was also conducted through the maximum parsimony method by using a bootstrap mode; consistency index recorded was 0.921958 for all sites. In the phylogenetic tree, *Potentilla indica* from Shogran and Garhi Habibullah, *Fragaria nubicola* from Jabori, KundBangla, and Battal, *Fragaria vesca* from Balakot, reference species MH711106.1, KF873772.1, AF163500.1, AF163517.1, and MG236177.1 were placed together in clad 1. *Fragaria* × *ananassa* from Qalanderabad and Baffa and other reference species such as KX166972.1, MG235113.1, AF163520.1, MG235200.1, JN999221.1, AF163494.1, AF163498.1, MN601853.1, and KT695219.1 were together in clad 2, while *Fragaria* × *ananassa* Duch from Dodial was placed out of the group as it showed diversity (Figure 13).

### 3.2. Molecular Phylogenetic Analysis of *Fragaria* by Using the *rbclC* Marker

**Figure 14:**
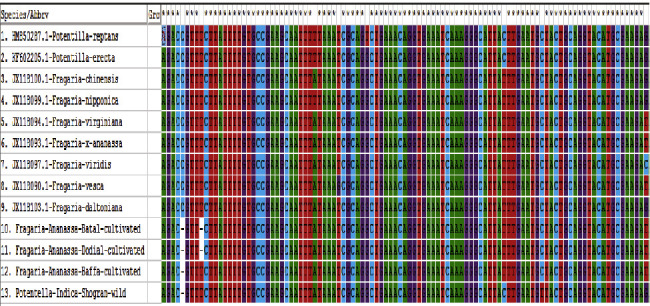
Basic local alignment of the selected *Fragaria* species (*rbclC*) marker with related reference sequences.

The *rbclC* sequence of *Fragaria* species was BLAST in NCBI (database) with related reference species and prepared in the FASTA format. According to the results, alignments ranged from 96% to 99.58% along with *Fragaria* × *ananassa* Duch (KY358226.1) which has 99.58% identity and 0.0 E-values with 95% query coverage. The sequences of selected *Fragaria* species and related reference sequences were aligned and trimmed by the removal of irregular nucleotide sequence than used for phylogenetic analysis (Figure 14).

**Figure 15:**
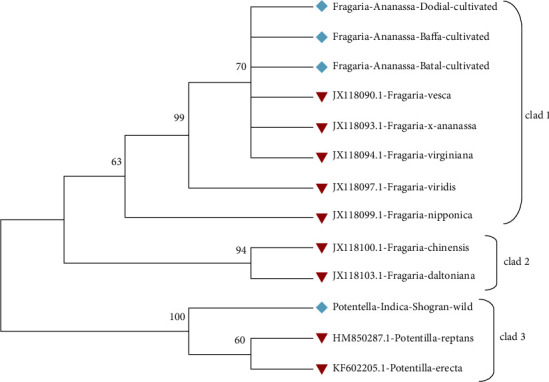
Phylogenetic tree analysis of *Fragaria* species (rbclC) with reference species through using the maximum likelihood method.

The phylogenetic tree constructed through the maximum likelihood method by using the bootstrap mode had a branch length of -1363.6855. The *Fragaria* × *ananassa* from Dodial, Baffa, and Batal and reference species JX118090.1, JX118093.1, JX18097.1, and JX118099.1 were found together in clad 1. *Fragaria chilonosis* (JX118100.1) and *Fragaria daltoniana* (JX118103.1) were placed in cladding 2, while *Potentilla reptans* (HM850287.1) and *Potentilla erecta* (KF602205.1) from Shogran were placed in clad 3 (Figure 15).

**Figure 16:**
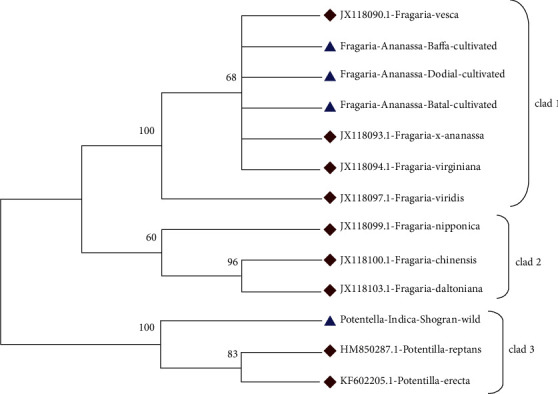
Phylogenetic tree of selected *Fragaria* (*rbclC*) species with related reference sequences through the neighbor-joining method.

The phylogenetic tree constructed with the neighbor-joining method had 0.07867073 branch lengths. *Fragaria* × *ananassa* Duch from Dodial, Baffa, and Battal and reference species JX228090.1, JX118093.1, JX118094.1, and JX118097.1 were placed together in clad 1.*Fragaria nipponica* (JX11099.1), *Fragaria chilonesis* (JX118100.1), and *Fragaria daltoniana* (JX118103.1) were placed in clad 2, whereas *Potentilla indicia*, *Potentilla reptans* (HM850287.1), and *Potentilla erecta* (KF602205.1) collected from Shogran were placed in clad 3 (Figure 16).

**Figure 17:**
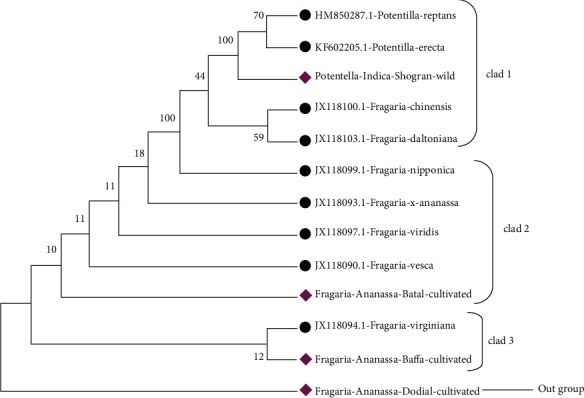
Phylogenetic tree analysis of the *Fragaria* species (*rbclC*) marker with related reference species through the maximum parsimony method.

The phylogenetic tree constructed by applying the maximum parsimony method contained a consistency index of 0.752598 for all sites. In the phylogenetic tree, *Potentilla indica* from Shogran, *Potentilla erecta* (KF602205.1), *Potentilla reptans* (HM850287.1), *Fragaria chilonesis* (JX118100.1), and *Fragaria daltoniana* (JX118103.1) are the part of clad 1. The *Fragaria* × *ananassa* Duch from Battal and reference species JX118099.1, JX118093.1, JX118097.1, and JX118090.1 were placed in clad 2. The *Fragaria* × *ananassa* from Baffa and *Fragaria virginiana* (JX118094.1) were placed in clad 3, while *Fragaria* × *ananassa* collected from Dodial showed diversity and therefore placed out of the group (Figure 17).

## 4. Conclusion and Recommendation

The result of this study showed that DNA barcoding is a useful technique for the identification and investigation of unknown species. Initially, we studied eight barcode markers among these two DNA barcode markers (ITS2, rbclC) which were successfully amplified and showed the significant result. The genetic diversity of selected Fragaria species showed similarities with related reference species. Furthermore, Potentilla indica from Shogran showed diversity which is the wild relative of Fragaria strawberry.

To fully understand genetic diversity, the following recommendations can be considered:

- More DNA markers should be used to understand the genetic diversity of Fragaria species
- DNA barcoding markers would be used against different populations for the identification of specific locus and genetic diversity within and between populations

## Figures and Tables

**Table 1:** GPS location of different regions of Mansehra, Pakistan.

S. no	Locations	Latitude (N)	Longitude (E)	Elevation (meter)
1	Jabori	34.6010	73.7645	1735
2	Garhi Habibullah	34.4012	73.4678	1790
3	Battal	34.6870	73.7456	1518
4	Baffa	34.5035	73.1001	1207
5	Shogran	34.6063	73.8504	2870
6	Balakot	34.5189	73.6345	1985
7	Qalandarabad	34.3022	73.3534	1193
8	Kund Bangla	34.3018	73.2158	2975
9	Dodial	34.1004	73.1972	1120
10	Chattar	34.4232	73.6873	2071

**Table 2:** Conditions of the selected markers and their sequences used in the current molecular study.

Sr.No	Primers	Primer name	Sequence	Melting temperature (°C)	References
1	ITS2-F ITS2-R	ITS-S2F ITS-S3R	5′ATGCGATACTTGGTGTGAAT 3′ 5′GACGCTTCTCCAGACTACAAT 3′	52	[17]
2	ITS-F ITS-R	ITS-F ITS-R	5′GCCGTTAAGACCAGGGAT 3′ 5′TGATTCACGGGATTCTGC 3′	50	[18]
3	rbcl-F rbcL-R	1f 724r	5′ATGTCACCACAAACAGAAAC3′ 5′TCGCATGTACCTGCAGTAGC 3′	56	[19]
4	rbclc-F rbCIC-R	HRM_rbcLCF HRM_rbcLCR	5′TAGACCTTTTTGAAGAAGGTTCTGT3′ 5′TGAGGCGGRCCTTGGAAAGTT 3′	76	[20]
5	rbclA- FrbclA-R	a-f a-r	5′ATGTCACCACAAACAGAGACTAAAGC 3′ 5′CTTCTGCTACAAATAAGAATCGATCTC 3′	63	[21]
6	matK-F MatK-R	2.1a forward 3.2 reverse	5′ATCCATCTGGAAATCTTAGTTC 3′ 5′ CTTCCTCTGTAAAGAATTC 3′	51	[22]
7	trnV-F trnV-R	trnV_UAC-E1F trnV_UAC-E1R	5′ GTAGAGCACCTCGTTTACAC 3′ 5′GTGTAAACGAGGTGCTCTAC 3′	56	[23]
8	ycf3-F ycf3-R	ycf3-E2F ycf3-E2R	5′GGCCGTGATCTGTCATTAC3′ 5′TTCCGCGTAATTTCCTTC3′	53.2	[23]

**Table 3:** Reagents of PCR and their volume.

Sr. No	Reagents	Quantity (*μ*l)
1	10 × Taq buffer	2.5
2	MgCl2	2.5
3	DNTPs	3.0
4	Primer (F & R)	2.0
5	Taq DNA Polymarase	0.5
6	Templet DNA	2.5
7	ddH2O	12
	**Total Volume**	**25**

## Data Availability

All the data are available within the manuscript.

## Supplementary Materials

Supplementary Materials The supplementary file to the manuscript contains details of the Agarose gel of extracted genomic DNA of selected *Fragaria* species, nucleotide sequences, and peaks of *Fragaria* species of the *ITS2* marker. The details of nucleotide sequence and peaks of *Fragaria* species of the *rbclC* marker are also added to it.
