# Supplementary material for: Molecular Characterization of Wild and Cultivated Strawberry (Fragaria × ananassa) through DNA Barcode Markers
Source: Genet Res (Camb). 2022 Oct 11;2022:9249561. doi: 10.1155/2022/9249561 (PMC9578897; doi:10.1155/2022/9249561)
Supplement: Supplementary Materials — The supplementary file to the manuscript contains details of the Agarose gel of extracted genomic DNA of selected Fragaria species, nucleotide sequences, and peaks of Fragaria species of the ITS2 marker. The details of nucleotide sequence and peaks of Fragaria species of the rbclC marker are also added to it. [file 9249561.f1.docx]

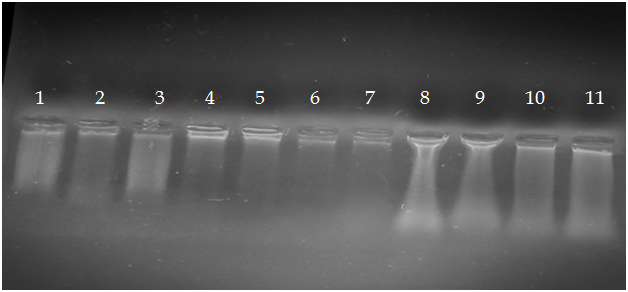
Figure 1: Agarose gel of extracted genomic DNA of selected*Fragaria* species.


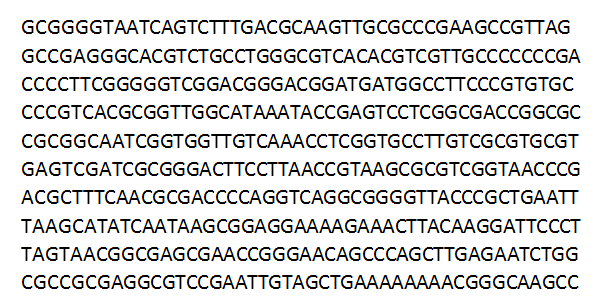
Figure 2: Sequence of *Fragaria* specie of *ITS2* marker.


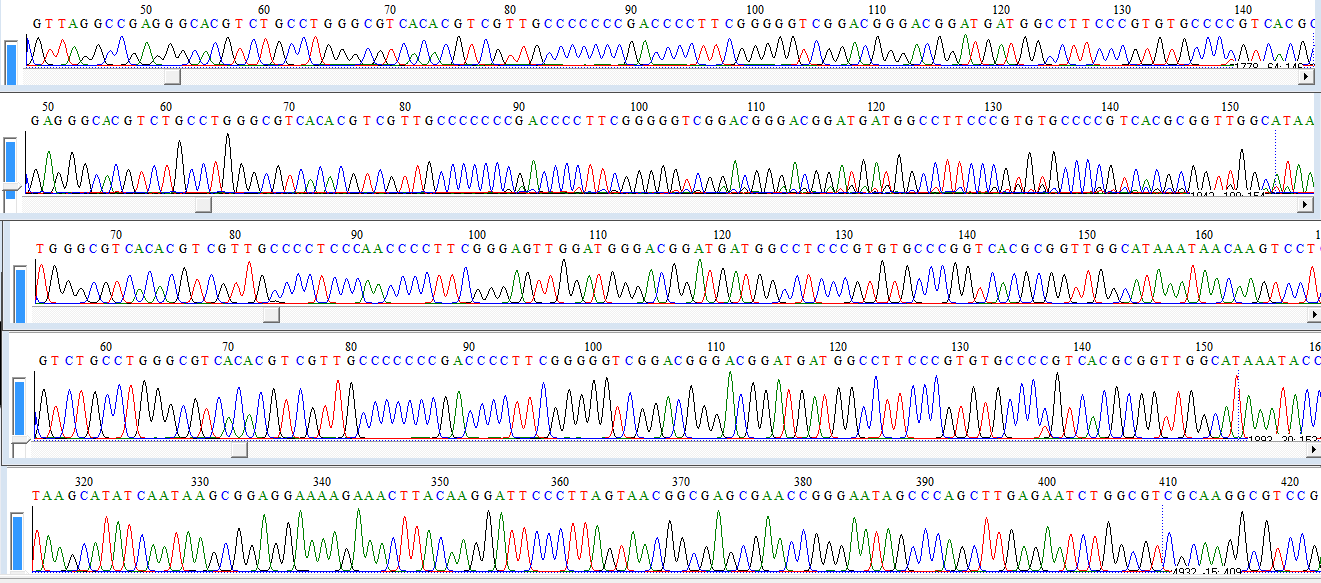
Figure 3: Nucteotide Sequences peaks of *Fragaria* species of *ITS2* marker.


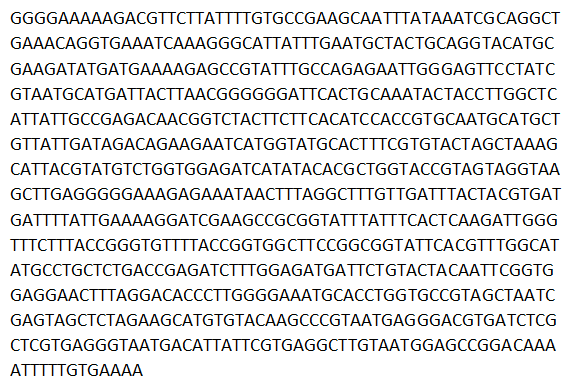


Figure 4: Nuclotides sequence of *Fragaria* specie of *rbclC* marker.


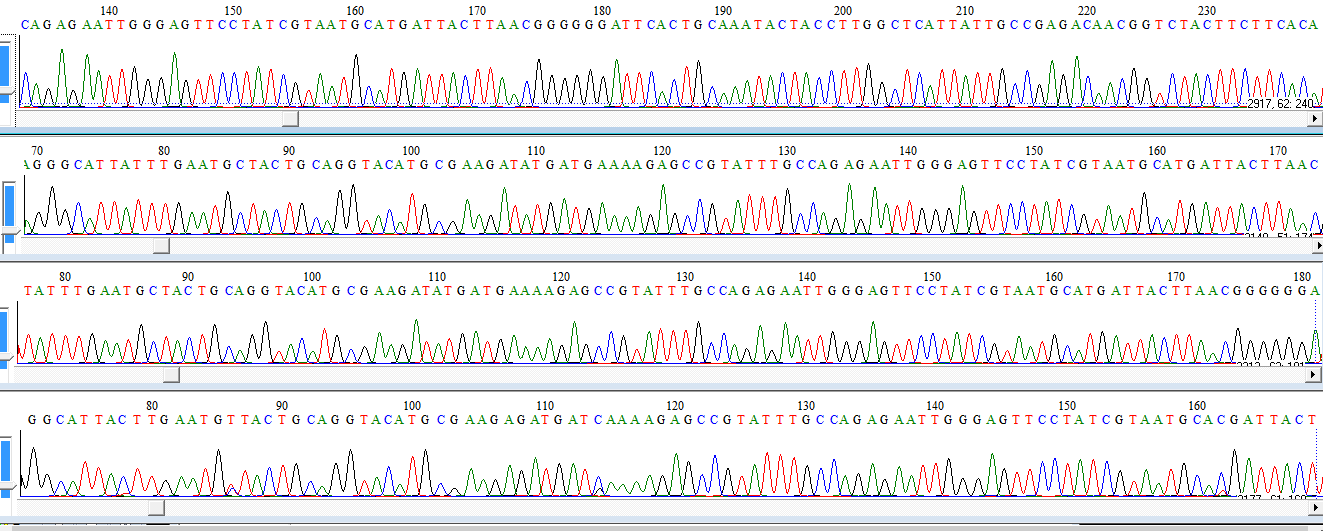
Figure 5: Nuclotide sequence peaks of *Fragaria* species of *rbclC* marker.
